# Influenza surveillance on ‘foie gras’ duck farms in Bulgaria, 2008–2012

**DOI:** 10.1111/irv.12368

**Published:** 2016-02-09

**Authors:** Atanaska Marinova‐Petkova, Georgi Georgiev, Todor Petkov, Daniel Darnell, John Franks, Ghazi Kayali, David Walker, Patrick Seiler, Angela Danner, Allison Graham, Pamela McKenzie, Scott Krauss, Richard J. Webby, Robert G. Webster

**Affiliations:** ^1^ Bulgarian Food Safety AgencySofiaBulgaria; ^2^ Bulgarian Society for the Protection of BirdsSofiaBulgaria; ^3^ St. Jude Children's Research HospitalMemphisTNUSA; ^4^ St. Jude Children's Research HospitalMemphisTNUSA

**Keywords:** ‘Foie gras’, avian influenza, ducks, European poultry, influenza surveillance, low‐pathogenicity avian influenza viruses

## Abstract

**Objectives:**

Ducks can shed and spread influenza A viruses (IAVs) while showing no disease signs. Our objective was to clarify the role of ‘foie gras’ ducks in the circulation of IAVs in Bulgaria.

**Methods:**

Monthly avian influenza surveillance was conducted on 63 ‘foie gras’ duck farms, 52 of which were surveyed throughout the study between November 2008 and April 2012. Virologic and serologic samples were collected and tested. During this time, wild bird samples were collected at major wild bird‐resting areas near the Black Sea coast and Danube River.

**Results:**

The study showed high isolation frequency of low‐pathogenicity avian influenza viruses. In the raising population (<75 days old), subtypes H3, H4, and H6 were detected monthly and H5 LPAIV, sporadically. Different subtypes (H1, H10, H11) were isolated from the fattening premises (75‐ to 100‐day‐old ducks), suggesting different routes of introduction. Only 6 of the 52 farms that were surveyed both virologically and serologically were influenza‐free throughout the study, possibly due to higher biosecurity measures implemented. No evidence of direct transmission of IAV from wild birds was found. Wild bird surveillance showed low isolation frequency of IAV. IAV prevalence of 0·55% for migratory ducks and 0·53% for migratory geese was estimated in November–December 2011 and January–February 2012, respectively, at two ornithologically important locations near the Black Sea coast.

**Conclusions:**

The ‘foie gras’ duck farms in Bulgaria are an optimal niche where Eurasian‐like IAVs are maintained and reassorted unapparent to farmers and veterinarians.

## Introduction

Wild aquatic birds are the major natural reservoir of influenza A viruses (IAVs).^1^ Extensive global influenza surveillance in migratory waterfowl has been conducted; however, few articles discuss surveillance in domestic or mule ducks.^2^, ^3^ Surveillance in farmed ducks raised for fatty liver (‘foie gras’) production in Bulgaria has been reported to the European Commission,^4^ but has not been previously described in detail.

The commercial poultry sector in Bulgaria includes mule ducks, hybrid ducks raised for ‘foie gras’ (FG) and meat production. Bulgaria has been a FG producer since 1960. In 2010, the export value reached 120 million Euros (150 million USD).^5^ In 2011, 5·5 million mule ducks were raised on 800 Bulgarian farms, making the country the second‐largest FG producer in Europe.^6^

The FG duck sector in Bulgaria has never experienced economic losses due to influenza‐like morbidity and mortality. Because of the constantly increasing number of mule ducks in the country, we investigated these ducks’ role as asymptomatic carriers in the ecology of IAV in the region. Because the country is located on important migratory routes connecting Western Siberia and Central and northern Europe with Africa, Bosphorus, and the Dardanelle straits,^7^, ^8^ we also conducted parallel surveillance in wild birds.

## Methods

### ‘Foie gras’ duck‐raising practices in Bulgaria and avian influenza surveillance on farms

**Figure 1:**
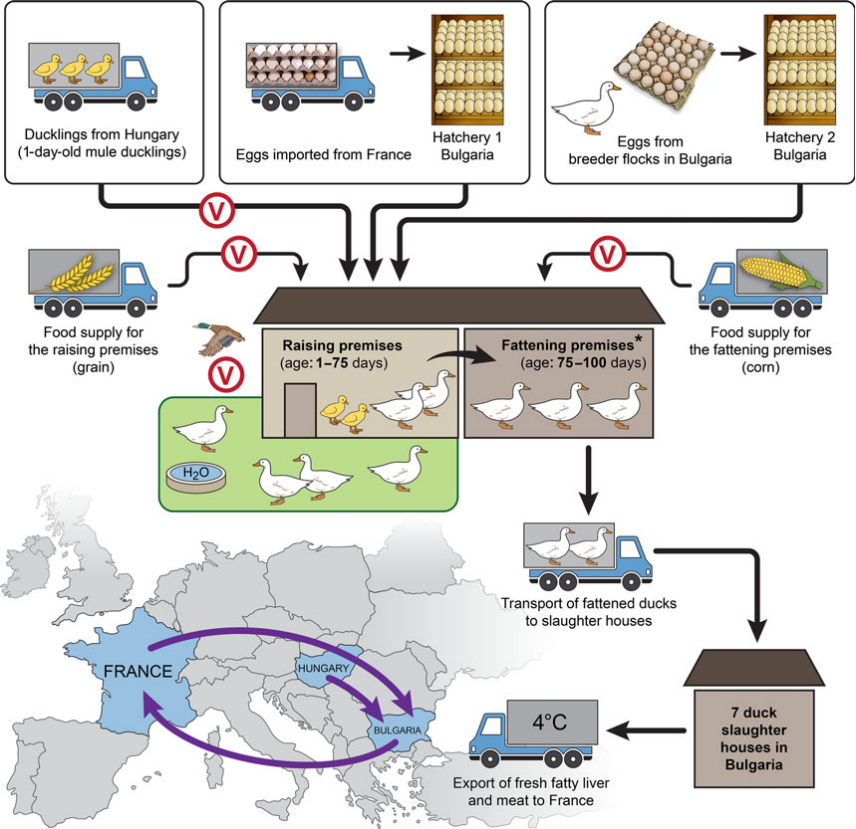
‘Foie gras’ duck‐raising cycle in Bulgaria. (*)Type I farms (shown in the illustration) consist of raising and fattening premises at the same location; the birds are directly transferred for fattening. Type II farms have the fattening premises at a different location, so the 75‐day‐old ducks must be transported by trucks to the fattening farm. All farms consist of multiple premises with mule ducks at different ages/stages. There is no ‘all‐in–all‐out’ principle: 1‐day‐old birds are introduced onto the farm as soon as space becomes available after flocks get moved for fattening. Circled V indicates steps at which we found a high risk of introduction of influenza A viruses onto the farms. The map shows the import and export of birds, fertile eggs, and duck products between Bulgaria and other European countries.

FG duck‐raising practices are illustrated in Figure 1. We conducted AI surveillance in the five regions of Bulgaria with the highest density of ducks during 4 winter–spring seasons between November 2008 and April 2012. During 3 of these seasons, the study focused on the raising duck populations (1–75 days old) considered to be at risk of acquiring IAV during their contact with wild birds while spending the day outside the farms (Figure 1). Ducks (75–100 days old) were actively fattened in closed premises, at which stage their risk of IAV direct transmission from wild birds was lower.

**Figure 2:**
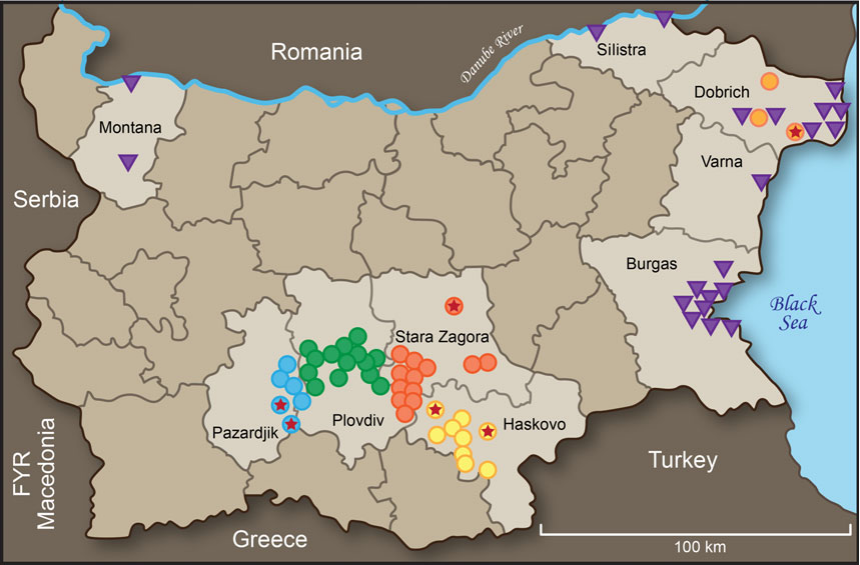
Map of Bulgaria and locations of sample collection (November 2008–April 2012). Regions important for the study appear in a lighter color. The circles are in region‐specific colors and represent the locations of the mule duck farms. A star in the circle denotes an influenza‐free farm. The purple triangles denote where wild bird samples were collected.

**Figure 3:**
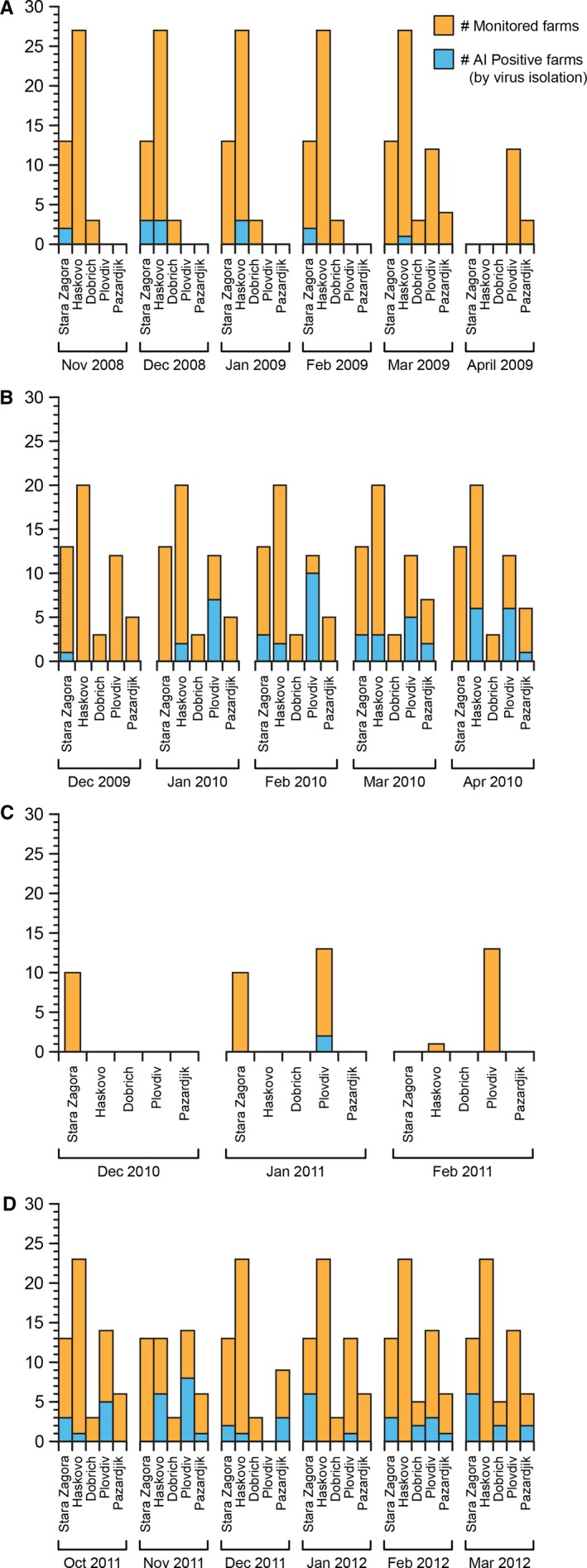
Number of influenza A‐positive farms and total number of monitored farms in Bulgaria during November 2008–April 2009 (A), December 2009–April 2010 (B), December 2010–February 2011 (C), and October 2011–March 2012 (D).

We monitored 27 FG duck farms in Haskovo, 13 in Stara Zagora, 14 in Plovdiv, 6 in Pazardjik, and 3 in Dobrich (approximate locations shown in Figure 2) on a monthly basis during the surveillance periods shown in Figure 3. Fifty‐two of the 63 farms were surveyed throughout the study. Multiple flocks of different ages and raised in separate premises were sampled monthly from all farms in Plovdiv and Pazardjik and from two farms in Haskovo.

Because of a funding lapse, during the third surveillance season (December 2010–February 2011), we decreased the number of monitored farms and focused only on the fattening premises of 10 farms in Stara Zagora, 13 farms in Plovdiv, and 6 premises of the biggest farm in the Haskovo region (Figure 3).

A total of 4774 cloacal swabs (CS), 1774 fecal samples (FS), and 2130 serum samples (SS) were collected from mule ducks. Depending on the size of each flock, 3 to 10 CS and 1 or 2 FS were collected monthly from each premise during the surveillance periods shown in Figure 3, whereas SS (15 per flock) were only collected in March and April of 2010 from 52 of the farms. Each month, different duck flocks were sampled. To evaluate the risk of vertical viral transmission, we collected 126 virologic samples from the active (adult) breeder flocks and 107 virologic samples from the non‐active (juvenile) breeder flocks, as well as 40 virologic samples from ducklings at the two hatcheries in Bulgaria.

We conducted an epizootiologic survey among the farms’ veterinarians, including questions about the proximity of the farms to sites visited by migratory birds, source of 1‐day‐old ducklings, type and source of food for the raising/fattening ducks, transportation of food supplies to the farms, and disinfection practices.

### Wild bird surveillance

A total of 4990 wild bird samples [4681 FS, 195 CS of trapped birds, and 114 tissue samples (TS) of dead birds] were collected between November 2008 and March 2012 at multiple logistically accessible locations around the major wild bird‐resting areas near the Black Sea coast and Danube River^9^, ^10^, ^11^ (Figure 2). The number of samples collected at each location, bird types and species, and surveillance seasons are shown in Table S1.

### Specimen collection, storage, and processing

All CS and FS were collected with Dacron^®^ swabs and stored in 1 ml of glycerol transport medium^12^ at −80°C. Prior to testing, up to 5 CS or 2 FS from mule ducks, collected from the same age group, flock, and premise were pooled. Wild bird samples were tested individually. SS from mule ducks were stored at −20°C.

### Virus isolation

Each CS, FS, and TS (individual or pooled) was injected into the allantoic cavity of 10‐day‐old embryonated chicken eggs and tested for the presence of IAV in hemagglutination assays^13^ by using 1% chicken erythrocytes and the Directigen^™^ EZ Flu A+B (Becton Dickinson, Franklin Lakes, NJ, USA) rapid test.

### RNA extraction and virus subtyping

RNA was isolated from allantoic fluids positive for IAV by using QIAamp^®^ Viral RNA Mini Kit (Qiagen, Hilden, Germany). IAV subtypes were characterized by performing reverse transcription–polymerase chain reaction (RT–PCR) assays. Subtypes H1–H15 were determined as described by Lee *et al*.^14^, and N1 and N2, as described by Capua *et al*.^15^. Primer sequences for the detection of N3–N9 subtypes were kindly provided by the FAO Reference Laboratory on AI and Newcastle Disease, IZSVe, Padova, Italy.

### Viral genome sequencing and phylogenetic analysis

DNA sequencing was performed by the Hartwell Center for Bioinformatics and Biotechnology at St. Jude Children's Research Hospital, as described.^16^ HA and NA gene sequences obtained in this study were deposited in the Influenza Research Database and are available under GenBank accession numbers: KP714401–KP714478.

Phylogeny of the complete coding region of the HA and NA genes was reconstructed using the maximum‐likelihood method, Tamura–Nei model, and 1000 bootstrap replication test in MEGA 5.^17^ Reference sequences were retrieved from GenBank based on either BLAST comparison with the Bulgarian duck viruses or the subtype and geographic location of the reference isolate.

### Serologic tests

The sera from mule ducks were first screened for the presence of antibodies against the nucleoprotein of IAV by using FlockChek AI MultiS‐Screen Ab Test Kit (Idexx, Maine, USA). The ELISA‐positive sera were subtyped by hemagglutination inhibition (HI) testing^18^ against a panel of AI antigens including A/duck/Italy/1447/05(H1N1), A/duck/Ukraine/63(H3N8), A/Duck/Czech/56(H4N6), A/mallard/ Italy/3401/05(H5N1), A/Turkey/Massachusetts/65(H6N2), A/chicken/Italy/1067/V99(H7N1), and A/mule duck/Bulgaria/229/2010(H10N7). Antibody subtypes were confirmed with A/mule duck/Bulgaria/427/2010(H1N1), A/mule duck/Bulgaria/101/2010(H3N6), A/mule duck/Bulgaria/188/2010(H4N2), A/duck/Italy/775/04(H5N3), A/mule duck/Bulgaria/160/2010(H6N2), An HI titer of 8 was used as the cutoff point for positivity.

### Statistical analysis

We used chi‐square test on PASW v18 (IBM, Armonk, NY, USA) to compare the isolation frequency of influenza A viruses in FG ducks by region, sampling season, age, and type of operation.

## Results

### Frequency of IAV isolation from FG duck farms in Bulgaria

During the first surveillance season (November 2008–April 2009), 21 IAVs were isolated from 304 pools of CS and 283 individually tested FS. All isolated IAVs were from the Stara Zagora and Haskovo regions; however, the Plovdiv and Pazardjik regions were not enrolled in the study until the end of the first season (March 2009). During the second season (December 2009–April 2010), 100 IAVs were isolated from 570 CS and FS pools; infection was detected in all regions except Dobrich. During the third season (December 2010–February 2011), from the 54 CS pools collected from the fattening premises, we had three positive for IAV, representing 2 farms in Plovdiv region. During the last surveillance season (October 2011–March 2012), 104 IAVs were isolated from 846 CS and FS pools. All infected flocks were asymptomatic during AI infection, and their growth and weight were reported as being normal. No IAVs were detected in the fecal samples from newly hatched ducklings in the two hatcheries suggesting a relatively low risk of vertical transmission to farms. Frequency of IAV isolation in FG ducks varied by region (*P*‐value < 0·001) with 18% detected in Plovdiv, around 10% in Pazardjik and Stara Zagora, and around 6% in Dobrich and Haskovo. The highest isolation frequency was detected during the second surveillance season (17·5%, *P*‐value < 0·001). Influenza A was more common in the raising duck population (<75 days of age) than in older ducks with prevalence rates of 12·1% and 5·2%, respectively. No significant difference in isolation frequency was detected by operation type (Table S2).

### Frequency of IAV isolation from wild birds in Bulgaria

Only 5 IAVs were isolated from 4990 samples collected from wild birds during 2008–2012. Between November 11, 2011 and December 11, 2011, we conducted 11 sampling events at Atanasovsko Lake in Burgas, collecting a total of 361 FS from migratory ducks (Common shelduck, Mallard, Northern shoveler, and Eurasian teal) and isolating A/wild duck/Bulgaria‐Burgas/173‐3/2011(H4N6) and A/wild duck/Bulgaria‐Burgas/196‐2/2011(H1), resulting in IAV prevalence rate of 0·55% (95% CI 0·5–0·6) for the waterfowl for this period and location. Between January 11, 2012 and February 15, 2012, 570 FS from White‐fronted, Red‐breasted, and Bean geese were collected in the fields within the 10‐km zone west of Burgas Lake and Mandra Lake during 16 sampling events. A/wild goose/Bulgaria‐Burgas/211‐7/2012(H1), A/wild goose/Bulgaria‐Burgas/212‐4/2012(H10), and A/wild goose/Bulgaria‐Burgas/212‐5/2012(H10) were isolated from these samples, yielding a 0·53% (95% CI 0·49–0·57) IAV prevalence in the geese during this period.

### Influenza virus subtypes

**Figure 4:**
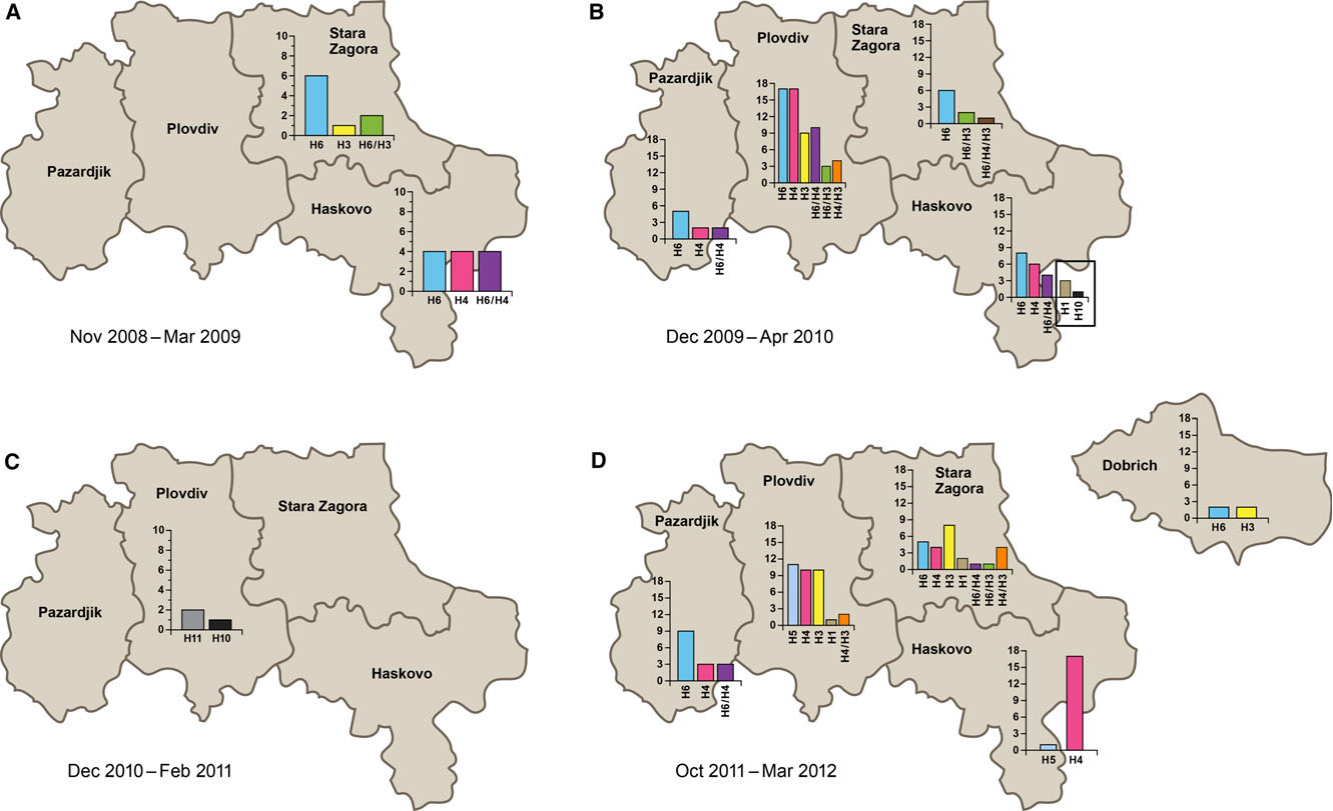
Influenza A virus subtypes detected in the ‘foie gras’ duck farms of the five monitored regions in Bulgaria during November 2008–April 2009 (A), December 2009–April 2010 (B), December 2010–February 2011 (C), and October 2011–March 2012 (D). Each IAV subtype is shown in a different color. HA subtypes are presented on the *x*‐axis. Dash between 2 HA subtypes represents their co‐isolation from the same pool of samples and same duck flock. Left *y*‐axis represents the number of isolated viruses per HA subtype.

During this study, IAVs of subtypes H6 (H6N2, H6N5, H6N6, and H6N8), H4 (H4N2, H4N6, and H4N8), and H3 (H3N2, H3N6, and H3N8) were frequently circulating in the raising mule duck population (Figure 4A, B and D). In contrast, only H1(H1N1 and H1N2), H10N7, and H11 viruses were isolated from the fattening mule duck population (Figure 4B,C). In 43 of 225 isolates, we detected a mix of 2 HA subtypes (Figure 4A, B and D); in five isolates, we detected only a second NA subtype. Most of the mixed subtypes were detected in the pools of samples, showing co‐circulation of 2 IAVs within the same flock; however, two single‐host fecal samples also contained 2 IAV subtypes.

Viruses from subtype H3 were not isolated in Haskovo or Pazardjik. In 2012, H6 and H3 viruses were detected from 2 of the farms in Dobrich after they had previously been influenza‐free (Figure 4D). In October 2011–November 2011, we detected the circulation of low‐pathogenicity avian influenza virus (LPAIV) H5N2 (HA cleavage site PQRETRGLF) in multiple flocks on 4 farms from three different locations in Plovdiv and LPAIV H5N8 (HA cleavage site PQRETRGLF) on a farm in Haskovo (Figure 4D). All H5 isolates from Plovdiv were obtained from ducks imported from Hungary as 1‐day‐old birds, suggesting the possible way of virus introduction. AI was not detected in the active duck breeders. AIV H6N5 was isolated once from the juvenile breeders, which spend the days outside on the raising duck farms.

IAV HA subtypes detected on 52 farms surveyed both virologically and serologically are shown in Table 1. IAVs were not isolated from farms surveyed during the first, or the first and last season only; however, they were not serosurveyed. At some farms, IAVs were isolated repeatedly throughout the season, whereas at others, only once (Table 1). Several farms had multiple IAV subtypes circulating during the same season.

**Table 1:** Comparison of subtypes of influenza A antibodies detected in duck sera from 52 farms in Bulgaria and isolated influenza A virus subtypes. Subtypes separated by a comma were isolated from or detected in different flocks. Subtypes separated by a slash were isolated from or detected in the same individual or pooled sample

Region	Farm	Viruses isolated November 08–March 09	Viruses isolated December 09–April 10	Antibodies detected March 10	Antibodies detected April 10	Viruses isolated October 11–March 12
Stara Zagora	1	–	–	–	–	H6
	2	–	–	α‐H6	α‐H4/H6	H3, H3/H4
	3	H3, H6	–	α‐H6	α‐H6	–
	4	H3/H6	H6	α‐H4/H5/H6	α‐H4/H6	H3
	5	H6	H3, H6, H3/H4/H6, H3/H6	–	α‐H6	H3, H3/H4
	6	H6	–	α‐H4/H5/H6	α‐H3/H4/H5	H3, H6
	7	H6	H6	α‐H5	α‐H4/H5/H6	H6
	8	–	H6	α‐H5	α‐H3/H4/H5	–
	9	–	–	α‐H6	α‐H6	H6, H6/H3
	10	H6	H6	α‐H6	α‐H4/H6	H1
	11	–	–	α‐H6	α‐H4/H6	H4, H4/H6
	12	H6	–	α‐H6	α‐H6	H4
	13	–	–	–	–	–
Haskovo	1	–	H4, H6	α‐H4	α‐H5	–
	2	–	–	–	–	–
	3	H6	–	α‐H6	–	N/A
	4	H6	H4/H6	–	α‐H3/H6	H4
	5	–	H1*, H10*	α‐H6*	–	–
	6	–	H6	α‐H6	α‐H4/H6	–
	7	–	–	–	–	–
	8	–	H4	α‐H1	α‐H3/H4	H5N8
	9	H4, H4/H6	–	–	–	–
	10	–	H1*	–	–	–
	11	–	–	α‐H6	α‐H6	–
	12	H4	H6	–	α‐H5/H6	H4
	13	H6	–	α‐H6	α‐H6	H4
	14	H4/H6	H6	α‐H6	α‐H6	H4
	15	–	H6	α‐H6	α‐H6, H4/H6, H5/H6, H4/H5/H6	H4
	16	–	–	α‐H6	–	–
	17	H4	–	–	–	–
Plovdiv	1	–	H3	α‐H4	α‐H3/H4	
	2	–	–	α‐H5/H6	α‐H4/H5/H6	H1
	3	–	H6	α‐H4	α‐H4/H6	–
	4	–	H4/H6	α‐H6	α‐H6	H4
	5	–	H4, H6, H4/H6	α‐H6	α‐H6, H6	H5N2
	6	–	H4, H6, H3/H4	α‐H6	–	H5N2
	7	–	H3, H6, H4/H6	α‐H4	α‐H3/H4/H6	H3
	8	–	H4, H6	α‐H4/H6	α‐H3/H4/H6	H4
	9	–	H6, H4/H6	–	α‐H4/H6	H4, H4/H3
	10	–	H3, H4, H6	α‐H4/H6	α‐H4/H6	H3, H4
	11	–	H3, H4/H6	α‐H3/H4	–	H5N2
	12	–	H4, H6	α‐H6	α‐H6	H5N2
	13	–	H3	α‐H4/H6	α‐H3/H4/H6	–
Pazardjik	1	–	H6	–	–	
	2	–	–	α‐H6	–	H6
	3	–	–	–	–	
	4	–	H4, H6	–	α‐H6	H4, H6, H4/H6
	5	–	–	–	–	
	6	–	–	–	–	H6
Dobrich	1	–	–	–	–	H6
	2	–	–	–	–	–
	3	–	–	–	–	H3

*Subtypes isolated from or detected in ducks from fattening premises (75–100 days old).

### Influenza A antibody detection

Of 2130 sera tested by ELISA, 1260 (59%) had antibodies against IAV. HI titers of the positive samples for all subtypes determined were within the range 1/16‐1/64.

In 635 individual SS, we detected antibodies against 2 or 3 IAV subtypes; this was consistent among the sera collected from the same duck flock. However, because the panel of HI antigens used was limited to only 7 HA subtypes, the presence of antibodies against the other nine subtypes cannot be ruled out. In several flocks, some ducks were shedding virus cloacally, while others had antibodies against the same IAV subtype. Serology results showed that ducks from farms in Stara Zagora, Haskovo, and Plovdiv have been exposed to H5 viruses during the second season despite our failing to isolate them. Only 6 farms in this study could be considered influenza‐free based on both negative virus isolation and antibody detection (Table 1).

### Epizootiologic survey

Low biosecurity measures were reported from most FG farms. In contrast to the infected farms, the influenza‐free farms were in isolated areas with limited traffic. All six had restricted access, underwent regular disinfection, and were in close proximity (<5 km) to large bodies of water where migratory birds stop over (Tundja River, Maritsa River, Black Sea). The raising ducks in these farms also spend days in open‐aired backyards.

All six influenza‐free farms were buying 1‐day‐old ducklings only from the local hatcheries and either had their own vehicles to transport food to the farm or were producing their own food. Most infected farms were hiring a transportation company for food delivery, and many farms were sharing the same vehicles. On all farms, the fattening ducks were fed semi‐boiled corn, whereas the raising population was fed combined, granulated fodders manufactured for growing ducks. The corn and the granules were supplied by different producers.

### Phylogenetic analysis of the HA and NA genes

**Figure 5:**
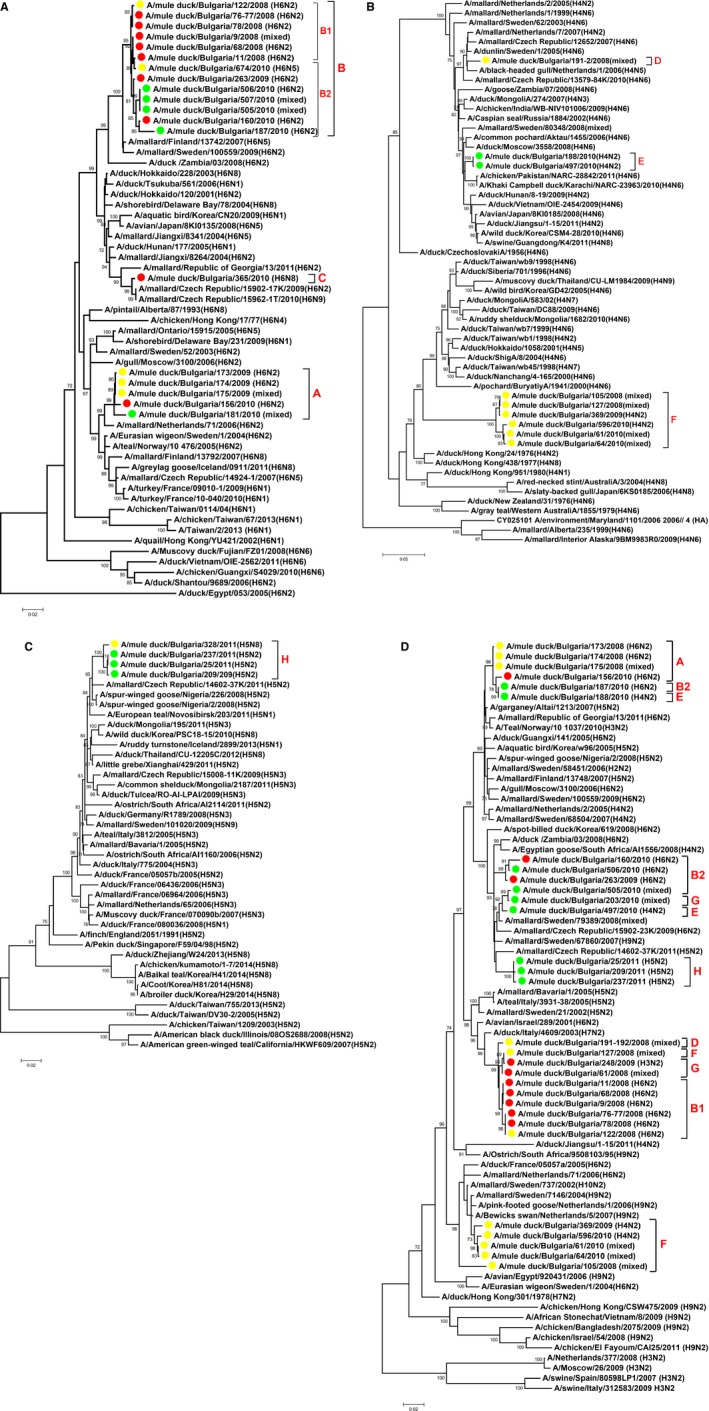
Phylogenetic relationships of HA gene of H6 IAVs (panel A); HA gene of H4 IAVs (panel B); HA gene of LPAIVs H5 (panel C); and NA gene of N2 IAVs (panel D) isolated from mule ducks in Bulgaria. Numbers at the branches indicate bootstrap values; only values >70 are shown. Red circles indicate viruses isolated in Stara Zagora; green circles, Plovdiv; and yellow circles, Haskovo.

Phylogenetic analysis of the HA gene of 19 H6 viruses showed at least three separate introductions of this subtype in the country (Figure 5A). Five Bulgarian H6 viruses from 2009–2010 share a common ancestor with a large cluster of 2004–2011 isolates from wild waterfowl from the Netherlands, Sweden, Norway, Finland, Iceland, Czech Republic and France (Figure 5A, group A). Thirteen of the viruses cluster together and share a common ancestor with mallard H6 isolates from Finland and Sweden from 2007 and 2009 (Figure 5A, group B). A/mule duck/Bulgaria/365/2010(H6) represents a separate introduction from mallards from the Czech Republic (Figure 5A, group C). The H6 viruses circulating in Plovdiv, Stara Zagora, and Haskovo in 2008–2010 are phylogenetically related, suggesting viral transmission among many farms after single introductions from wild waterfowl.

Our H4 phylogenetic tree reveals three different H4 lineages circulating in the mule ducks in Bulgaria (Figure 5B). One of them is closely genetically related to viruses from wild waterfowl isolated in the Netherlands, Sweden, or Czech Republic (Figure 5B, group D). Other H4 viruses, isolated in 2010 in Plovdiv region, share a common ancestor with poultry isolates from Pakistan (2010–2011) and are closely related to avian and swine isolates from Eastern Asia (2008–2011) and wild duck isolates from Russia (2006–2008) (Figure 5B, group E). A/duck/Jiangsu 1‐15/2011 (H4N2) also falls into this cluster; it appeared to be the closest genetic relative for 4 of the internal genes of the highly pathogenic avian influenza virus (HPAIV) H5N8 that circulated in Korea in 2014^19^ and then spread to Europe and North America. Its HA gene has only five amino acids differing from those of the Bulgarian mule duck isolates 188/2010 and 497/2010. The third lineage, circulating in Haskovo in 2008–2010, forms a distinct phylogenetic group that shares a common ancestor with a large cluster of viruses from Taiwan, China, Mongolia, Thailand, Japan, Korea, and Siberia (Figure 5B, group F). The closest relative to all six Bulgarian isolates in this group is A/duck/Taiwan/wb7/1999 (H4N6), with only 89–90% identity.

The H3 phylogenetic analysis shows that the viruses isolated in 2010 from farms in Plovdiv are closely related to those isolated in 2008–2009 in the neighboring region of Stara Zagora, and we labeled them all as group G (Figure S1). All Bulgarian H3 viruses in the tree are genetically related to wild waterfowl IAVs isolated in the Netherlands, Sweden, and Switzerland between 2001 and 2006.

The LPAI H5 phylogenetic analysis shows that the H5N8/2011 isolate from Haskovo clusters with the three H5N2/2011 circulating in Plovdiv (Figure 5C). All four Bulgarian H5 isolates (group H) share a common ancestor with H5N2 viruses from spur‐winged geese isolated in 2008 in Nigeria but are also closely related to a mallard H5N2 isolate from the Czech Republic (2011).

The most common NA subtype isolated in this study was N2; its phylogenetic relationships showed multiple introductions in the mule duck population in Bulgaria (Figure 5D). Parallel analysis of the N2 and HA gene trees revealed possible reassortment within and among the IAV subtypes. The N2 gene of the H6N2 isolates from Stara Zagora and Haskovo from 2008 (Figure 5D, subgroup B1) is genetically related to the N2 of H4 and H3 isolates from the same year and locations (Figure 5D, groups D, F, G): they share common ancestor with A/duck/Italy/4609/2003 (H7N2). However, the H6N2 viruses circulating in Stara Zagora and Plovdiv in 2009–2010 (Figure 5D, subgroup B2) have NA genes related to isolates from Sweden and the Czech Republic (2008–2009) and that cluster with N2 genes of H3 and H4 Bulgarian isolates from 2010 (Figure 5D, groups E, G). Five of six isolates from the Taiwanese‐like H4 distinct lineage (Figure 5D, group F) have genetically similar N2 genes, related to H9N2 wild bird isolates from Sweden and the Netherlands, which were circulating between 2004 and 2007.

A/mule duck/Bulgaria/187/2010(H6N2), (group B2), A/mule duck/Bulgaria/188/2010(H4N2) (group E), and A/mule duck/Bulgaria/497/2010(H4N2) (group E) were isolated from the same farm in Plovdiv region. Isolates 187 and 188 were from the same flock of 67‐day‐old ducks sampled on February 11, 2010, whereas isolate 497 was from 19‐day‐old ducks sampled on March 26, 2010. The N2 tree showed that the co‐circulating H6 and H4 isolates in February have identical N2 genes (Figure 5D). The HA gene of the 2 H4N2 isolates is also identical, but their N2 genes fall into different clusters, showing possible interactions with a third IAV genotype on the farm. The N6 and N8 phylogenetic analyses showed multiple introductions into Bulgaria and close genetic relationship to wild bird viruses isolated in Sweden, the Netherlands, and the Czech Republic (Figures S2 and S3).

## Discussion

Our results indicate that wild waterfowl migrating to northwest Europe are the closest genetic relatives of most IAVs isolated from mule ducks in Bulgaria. The existing migratory route connecting northwest Europe and Bulgaria^11^, ^20^ together with the common outdoor duck‐raising practice offers ideal conditions for direct viral transmission between wild birds and FG ducks. However, the low IAV prevalence in the wild bird samples in Bulgaria between 2008 and 2012 and the miniscule geographic overlap between the major duck‐raising regions and the main resting areas for migratory birds do not directly support this hypothesis. Furthermore, all influenza‐free farms were near areas visited by migratory birds, suggesting again that direct contact with wild waterfowl was not a major part of the process of infecting farms with influenza.

LPAI H5 infection on several mule duck farms in the same geographic area in France (all in January 2005) was attributed to separate virus introductions from wild ducks.^3^ The Bulgarian LPAI H5 viruses from 2011 clearly share a common ancestor (Figure 5D) despite their occurrence in different geographic regions, suggesting that maybe different factors play key role in the AI infection on the duck farms in Bulgaria and France.

Close phylogenetic relationship among isolates from different farms and regions in Bulgaria shows a large spread of viruses after single introductions and may be due to the extremely low biosecurity measures on most FG farms. IAVs have most likely been spread among the farms by vehicles transporting live ducks and food, shared by many of the farms but not by the six influenza‐free farms. This hypothesis could also explain why the IAV subtypes circulating within the fattening duck flocks (H1, H10, and H11) were different from those found in the raising duck flocks (mainly H6, H3, H4), suggesting different methods of introduction. In fact, the type and sources of food for the raising and fattening populations are among the main differences between them.

Among the FG‐producing countries in Europe, H5 infection in fattening ducks has been reported annually between 2005 and 2012 by France^3^, ^4^ and Belgium (except in 2007 and 2009).^4^ In our study, anti‐H5 antibodies were detected on 8 farms in March–April 2010, but no H5 viruses were isolated during that surveillance season. Possible reasons could be that not all flocks on the farms were sampled or unsuccessful virus isolation if the ducks had stopped shedding virus by the time of sampling**.**

The exact route of introduction of viruses from East Asia remains unclear and probably involved overlapping migratory flyways. The Taiwanese‐like H4N2 lineage circulating in Haskovo might have been established in Bulgaria after reassortment between an Asian H4 strain and an H9N2 wild bird virus from Sweden/the Netherlands. The H4 gene of this lineage has low identity with its closest relatives in GenBank, possibly due to a much‐earlier introduction in Bulgaria, followed by evolution within the local duck population.

The recent emergence of H7N9, H6N1, and H10N8, which have low‐pathogenic characteristics for avian hosts, but can cause human infections, highlights the need for continuous LPAIV surveillance. Additionally, the widespread of highly pathogenic H5 viruses from clade 2.3.4.4 in 2014–2015 from Asia to Europe and North America^21^ reinforced the importance of biosecurity at poultry farms. The results of our study raise serious concerns that the introduction of HPAIV virus into FG duck farms in Bulgaria or in Europe, in general, will affect many farms, with eradication being difficult and costly.

## Conflicts of interest

All authors declare that they have no competing interests to report.

## Supporting information


**Figure S1.** Phylogenetic relationships of HA gene of H3 IAVs isolated from mule ducks in Bulgaria.

**Figure S2.** Phylogenetic relationships of NA gene of N6 IAVs isolated from mule ducks in Bulgaria.

**Figure S3.** Phylogenetic relationships of NA gene of N8 IAVs isolated from mule ducks in Bulgaria.

**Table S1.** Sample collection sites and number of specimens collected from wild birds in Bulgaria (December 2008–March 2012).

**Table S2.** Statistical analysis on frequency of influenza A virus isolation from ‘Foie Gras’ ducks in Bulgaria, 2008–2012.
