# Supplementary figures and images for: Influenza surveillance on ‘foie gras’ duck farms in Bulgaria, 2008–2012
Source: Influenza Other Respir Viruses. 2016 Feb 9;10(2):98–108. doi: 10.1111/irv.12368 (PMC4746559; doi:10.1111/irv.12368)

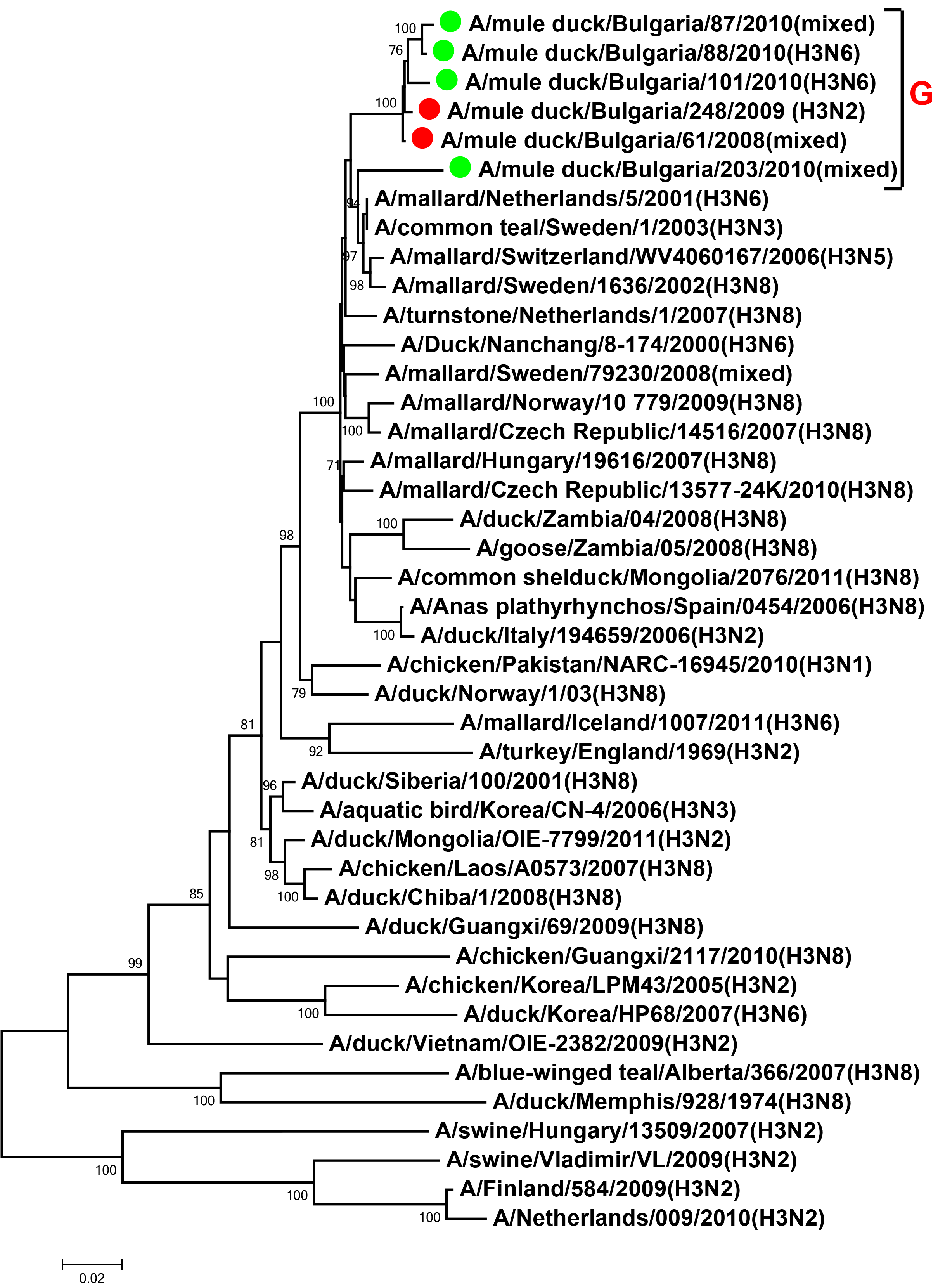

Supplement: Supplementary file 1 — Figure S1. Phylogenetic relationships of HA gene of H3 IAVs isolated from mule ducks in Bulgaria. [file IRV-10-098-s001.tif]

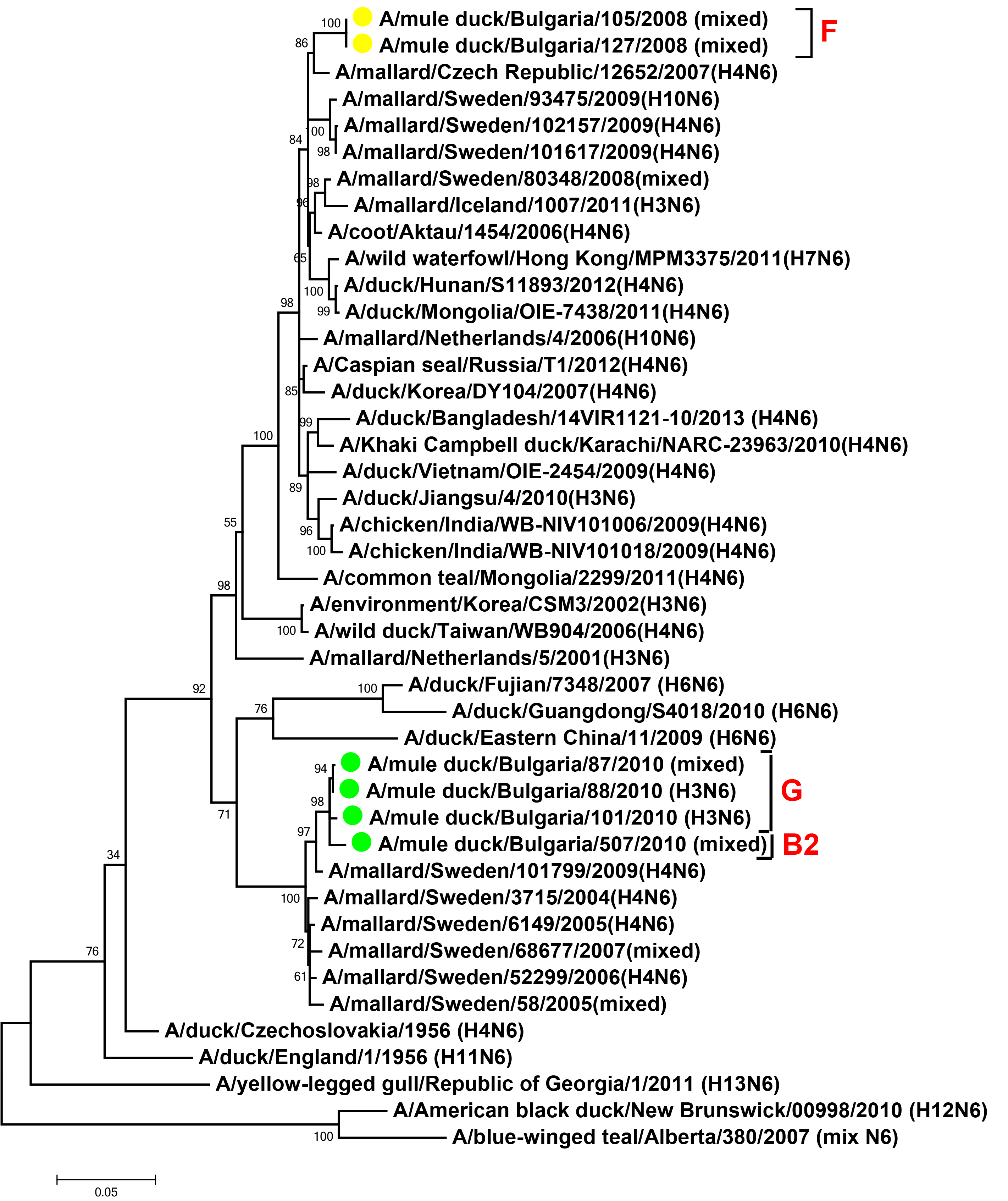

Supplement: Supplementary file 2 — Figure S2. Phylogenetic relationships of NA gene of N6 IAVs isolated from mule ducks in Bulgaria. [file IRV-10-098-s002.tif]

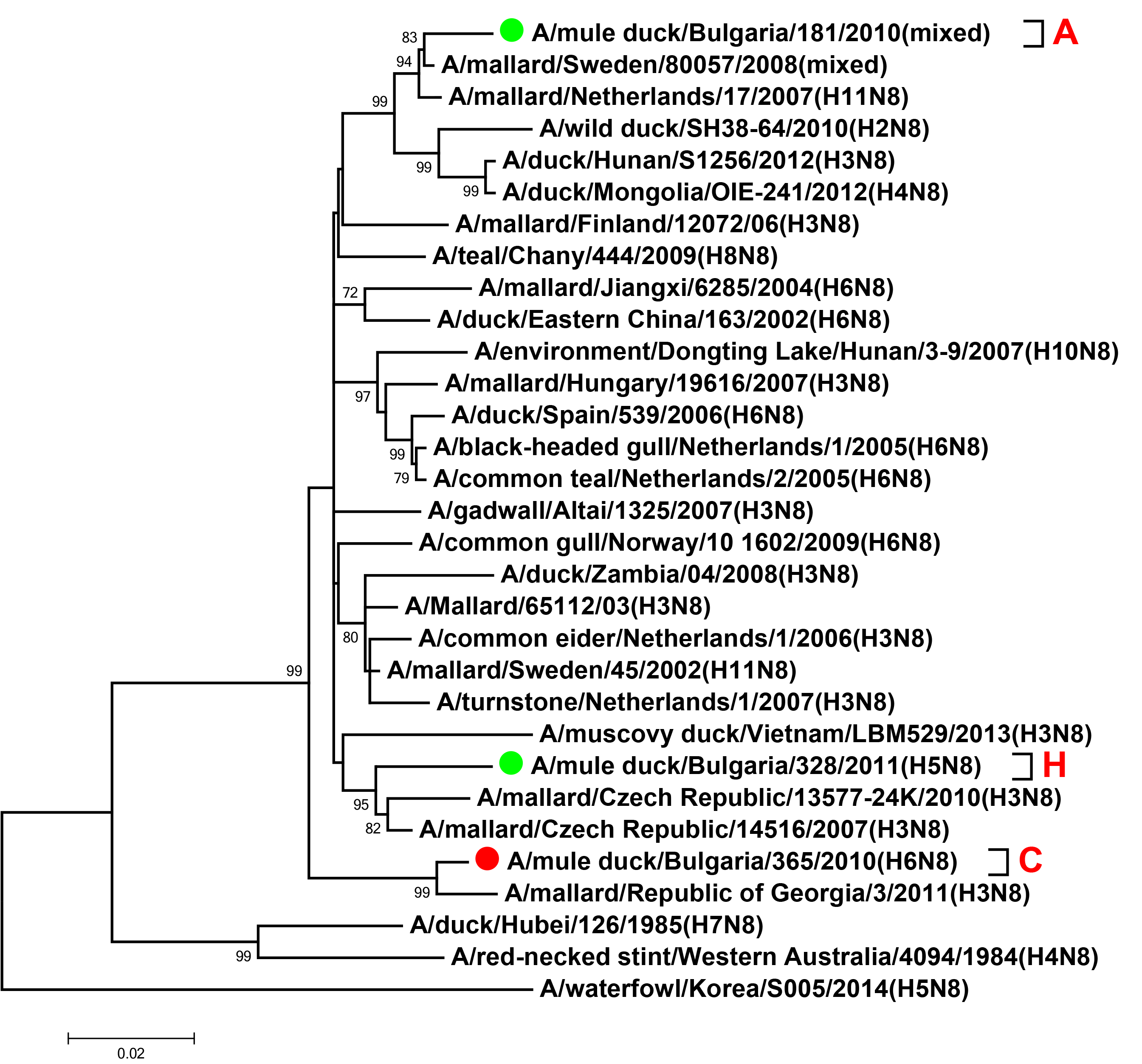

Supplement: Supplementary file 3 — Figure S3. Phylogenetic relationships of NA gene of N8 IAVs isolated from mule ducks in Bulgaria. [file IRV-10-098-s003.tif]
