# Supplementary material for: Influenza surveillance on ‘foie gras’ duck farms in Bulgaria, 2008–2012
Source: Influenza Other Respir Viruses. 2016 Feb 9;10(2):98–108. doi: 10.1111/irv.12368 (PMC4746559; doi:10.1111/irv.12368)
Supplement: Supplementary file 4 — Table S1. Sample collection sites and number of specimens collected from wild birds in Bulgaria (December 2008–March 2012). [file IRV-10-098-s004.docx]

Supplementary Table 1. Sample collection sites and number of specimens collected from wild birds in Bulgaria (December 2008 – March 2012).

^a^White-fronted goose (*Anser albifrons*), Red-breasted goose (*Branta ruficollis*), Bean goose (*Anser fabalis*), Mallard (*Anas platyrhynchos*), Common Shelduck (*Tadorna tadorna*) , Gadwall (*Anas strepera*), Eurasian teal (*Anas crecca*), Northern shoveler (*Anas clypeata*), Whooper swan (*Cygnus cygnus*)

^b^European herring gull (*Larus argentatus*), Lesser black-backed gull (*Larus fuscus*), Black-headed gull (*Chroicocephalus ridibundus*), Yellow-legged gull (*Larus michahellis*), Slender-billed gull (*Chroicocephalus genei*), Little gull (*Hydrocoloeus minutus*), Great cormorant (*Phalacrocorax carbo*), Pygmy cormorant (*Microcarbo pygmeus*), Common tern (*Sterna hirundo*)

^c^Glossy ibis (*Plegadis falcinellus*), Ruff (*Philomachus pugnax*), White stork (*Ciconia ciconia*), Common redshank (*Tringa totanus*), Pied avocet (*Recurvirostra avosetta*), Grey heron (*Ardea cinerea*), Eurasian woodcock (*Scolopax rusticola*)

^d^Eurasian coot (*Fulica atra*), Little grebe (*Tachybaptus ruficollis*), pelican (*Pelecanus onocrotalus*), pink pelican (*Pelecanus rufescens*)

^e^Sand martin (*Riparia riparia*), European serin (*Serinus serinus*), Eurasian siskin (*Carduelis spinus*), Eurasian linnet (*Carduelis cannabina*), Eurasian skylark (*Alauda arvensis*), White wagtail (*Motacilla alba*), Yellow wagtail (*Motacilla flava*), European goldfinch (*Carduelis carduelis*), European greenfinch (*Chloris chloris*), Eurasian reed warbler (*Acrocephalus scirpaceus*), Lesser whitethroat (*Sylvia curruca*), Sedge warbler (*Acrocephalus schoenobaenus*), Red-backed shrike (*Lanius collurio*), Barn swallow (*Hirundo rustica*), Red-rumped swallow (*Cecropis daurica*), European bee-eater (*Merops apiaster*), Great grey shrike (*Lanius excubitor*), Northern wheatear (*Oenanthe oenanthe*), Common swift (*Apus apus*), Orphean warbler (*Sylvia hortensis*), Willow sparrow (*Passer hispaniolensis*), Great tit (*Parus major*), Song thrush (*Turdus philomelos*). (Last two are the only non-migratory passerine birds)

^f^Common buzzard (*Buteo buteo*), Common kestrel (*Falco tinnunculus*), Golden eagle (*Aquila chrysaetos*), Eurasian goshawk (*Accipiter gentilis*), Owl (*Bubo bubo*), Little owl (*Athene noctua*), Long-eared owl (*Asio otus*).

| Sampling period | Sample collection site | | Types of birds | | | | | |
| --- | --- | --- | --- | --- | --- | --- | --- | --- |
|  | Region | Location/  GPS coordinates | Waterfowl^a^ | Seabirds^b^ | Wading  birds^c^ | Other water  birds^d^ | Passerine  birds^e^ | Birds of  prey^f^ |
| Dec’2008–Mar’2009 | Dobrich | Durankulak Lake  43°41'03.2"N 28°33'29.7"E | 182 | - | - | - | - | - |
| Jan’2010 – Apr’2010 | Dobrich | Durankulak Lake  43°41'03.2"N 28°33'29.7"E | 441 | - | - | 6 | - | - |
|  |  | Shabla Lake  43°34'30.4"N 28°33'56.5"E | 96 | - | - | - | - | - |
|  |  | Senokos village  43°31'14.8"N 28°01'32.0"E | 50 | - | - | - | - | - |
|  |  | Balchik/Black Sea  43°24'22.0"N 28°09'22.1"E | 11 | - | - | - | - | - |
|  | Montana | Lom River mouth/Danube  43°49'57.1"N 23°14'32.2"E | 81 | - | - | - | - | - |
|  |  | Ogosta artificial lake  43°22'56.4"N 23°11'10.7"E | 81 | - | - | - | - | - |
|  | Silistra | Pozharevo & Dunavets villages/Danube  44°03'36.8"N 26°42'43.0"E | 22 | - | - | - | - | - |
|  |  | Silistra/Danube  44°07'24.8"N 27°16'29.1"E | 4 | - | - | - | - | - |
|  | Varna | Varna  43°12'31.8"N 27°56'16.8"E | 8 | - | 2 | - | - | 10 |
| Jan’2011– Dec’2011 | Burgas | Atanasovsko Lake  42°34'19.8"N 27°27'50.9"E | 469 | 16 | 23 | - | 299 | 11 |
|  |  | Burgas Lake  42°29'20.1"N 27°24'00.1"E | - | 193 | - | - | - | - |
|  |  | Mandra Lake & Poda Protected Area  42°26'26.1"N 27°26'19.3"E | 56 | 76 | 12 | 4 | 27 | - |
|  |  | Other important migratory bird areas  within the 25km perimeter around Burgas  42°30'46.7"N 27°27'36.7"E | 554 | 10 | 7 | 4 | 147 | 11 |
|  |  | Pomoriisko Lake  42°34'33.9"N 27°37'02.2"E | 73 | - | - | 19 | - | - |
|  |  | Poroi Art. Lake & Galabets village  42°44'32.1"N 27°34'42.1"E | - | 12 | 2 | 2 | 163 | - |
|  |  | Sozopol, St Ivan Island  42°26'12.6"N 27°41'17.5"E | - | 81 | - | - | - | - |
|  | Dobrich | Durankulak Lake  43°41'03.2"N 28°33'29.7"E | 398 | 7 | - | - | - | - |
|  |  | Shabla Lake  43°34'30.4"N 28°33'56.5"E | 99 | 3 | - | - | - | - |
|  |  | Tyulenovo village/Black Sea  43°29'38.8"N 28°35'05.5"E | 29 | - | - | - | - | - |
|  |  | Ezerets village  43°35'37.6"N 28°32'08.6"E | 50 | - | - | - | - | - |
|  |  | Dobrich  43°35'37.6"N 28°32'08.6"E | - | - | - | - | - | 6 |
|  | Varna | Varna  43°12'39.7"N 27°56'42.2"E | - | - | - | 4 | 1 | 11 |
| Jan’2012 – Mar’2012 | Burgas | Atanasovsko Lake  42°34'19.8"N 27°27'50.9"E | - | - | - | - | - | 1 |
|  |  | Burgas Lake  42°29'20.1"N 27°24'00.1"E | 54 | - | - | - | - | - |
|  |  | Mandra Lake & Poda Protected Area  42°26'26.1"N 27°26'19.3"E | 22 | - | - | - | - | - |
|  |  | Other important migratory bird areas  within the 25km perimeter around Burgas  42°30'46.7"N 27°27'36.7"E | 637 | - | - | 10 | - | 5 |
|  |  | Pomoriisko Lake  42°34'33.9"N 27°37'02.2"E | 362 | - | - | - | - | - |
|  |  | Poroi Art. Lake  42°44'32.1"N 27°34'42.1"E | 26 | - | - | - | - | - |
| Total number of samples collected | | | 3805 | 398 | 46 | 49 | 637 | 55 |
