# Supplementary material for: Influenza surveillance on ‘foie gras’ duck farms in Bulgaria, 2008–2012
Source: Influenza Other Respir Viruses. 2016 Feb 9;10(2):98–108. doi: 10.1111/irv.12368 (PMC4746559; doi:10.1111/irv.12368)
Supplement: Supplementary file 5 — Table S2. Statistical analysis on frequency of influenza A virus isolation from ‘Foie Gras’ ducks in Bulgaria, 2008–2012. [file IRV-10-098-s005.docx]

Supplementary Table 2. Statistical analysis on frequency of influenza A virus isolation from ‘Foie Gras’ ducks in Bulgaria, 2008-2012. Chi-square test on PASW v18 (IBM, Armonk, NY) was used to compare isolation frequency of influenza A viruses in FG ducks by region, sampling season, age, and type of operation.

| **Variable** | **No. tested pools of**  **samples (%^*^)** | **No. of**  **Influenza A-positive pools (%**^†^**)** | **P-value** |
| --- | --- | --- | --- |
| *Region*  Dobrich  Haskovo  Pazardjik  Plovdiv  Stara Zagora | 69 (3.4)  795 (39.1)  224 (11.0)  538 (26.4) 409 (20.1) | 4 (5.8)  52 (6.5)  24 (10.7)  97 (18.0)  43 (10.5) | <0.001 |
| *Season*  1  2  3  4 | 563 (27.7)  572 (28.1)  54 (2.7)  846 (41.6) | 21 (3.7)  100 (17.5)  3 (5.6)  96 (11.3) | <0.001 |
| *Age*  < 75 days  > 75 days | 1656 (83.6)  326 (16.4) | 200 (12.1)  17 (5.2) | <0.001 |
| *Operation type*  Breeder/Hatchery  Farm | 49 (2.4)  1986 (97.6) | 2 (4.1)  218 (11.0) | Not significant |
|  |  |  |  |
