# Supplementary material for: Influenza surveillance on ‘foie gras’ duck farms in Bulgaria, 2008–2012
Source: Influenza Other Respir Viruses. 2016 Feb 9;10(2):98–108. doi: 10.1111/irv.12368 (PMC4746559; doi:10.1111/irv.12368)
Supplement: Supplementary file 6 [file IRV-10-098-s006.docx]

Supplementary Figure 1. Phylogenetic relationships of HA gene of H3 IAVs isolated from mule ducks in Bulgaria. Numbers at the branches indicate bootstrap values; only values >70 are shown. Red circles indicate viruses isolated in Stara Zagora; green circles indicate Plovdiv.

Supplementary Figure 1. Phylogenetic relationships of NA gene of N6 IAVs isolated from mule ducks in Bulgaria. Numbers at the branches indicate bootstrap values; only values >70 are shown. Green circles indicate viruses isolated in Plovdiv and yellow circles, in Haskovo.

Supplementary Figure 1. Phylogenetic relationships of NA gene of N8 IAVs isolated from mule ducks in Bulgaria. Numbers at the branches indicate bootstrap values; only values >70 are shown. Red circles indicate viruses isolated in Stara Zagora; green circles, Plovdiv.
